# The impact of demographic and perceptual variables on a young adult’s decision to be covered by private health insurance

**DOI:** 10.1186/s12913-015-0848-6

**Published:** 2015-05-12

**Authors:** John Cantiello, Myron D Fottler, Dawn Oetjen, Ning Jackie Zhang

**Affiliations:** Department of Health Administration and Policy, College of Health and Human Services, George Mason University, 4400 University Drive, MS 1J3, Fairfax, 22030, VA USA; Department of Healthcare Management and Informatics, College of Health and Public Affairs, University of Central Florida, 4000 Central Florida Blvd, 32816 Orlando, FL USA; Department of Interprofessional Health Sciences and Health Administration, Seton Hall University, 400 South Orange Ave., 07079 South Orange, NJ USA

**Keywords:** Health insurance coverage, Young adults

## Abstract

**Background:**

The large number of uninsured individuals in the United States creates negative consequences for those who are uninsured and for those who are covered by health insurance plans. Young adults between the ages of 18 and 24 are the largest uninsured population subgroup. This subgroup warrants analysis. The major aim of this study is to determine why young adults between the ages of 18 and 24 are the largest uninsured population subgroup.

**Methods:**

The present study seeks to determine why young adults between the ages of 18 and 24 are the largest population subgroup that is not covered by private health insurance. Data on perceived health status, perceived need, perceived value, socioeconomic status, gender, and race was obtained from a national sample of 1,340 young adults from the 2005 Medical Expenditure Panel Survey and examined for possible explanatory variables, as well as data on the same variables from a national sample of 1,463 from the 2008 Medical Expenditure Panel Survey.

**Results:**

Results of the structural equation model analysis indicate that insurance coverage in the 2005 sample was largely a function of higher socioeconomic status and being a non-minority. Perceived health status, perceived need, perceived value, and gender were not significant predictors of private health insurance coverage in the 2005 sample. However, in the 2008 sample, these indicators changed. Socioeconomic status, minority status, perceived health, perceived need, and perceived value were significant predictors of private health insurance coverage.

**Conclusions:**

The results of this study show that coverage by a private health insurance plan in the 2005 sample was largely a matter of having a higher socioeconomic status and having a non-minority status.

In 2008 each of the attitudinal variables (perceived health, perceived value, and perceived need) predicted whether subjects carried private insurance. Our findings suggest that among those sampled, the young adult subgroup between the ages of 18 and 24 does not necessarily represent a unique segment of the population, with behaviors differing from the rest of the sample.

## Background

### Introduction

The rising costs of medical services and insurance premiums are making health care unaffordable and inaccessible for many Americans. A recent U.S. Census Bureau report indicated that there were 42 million uninsured Americans living in the United States in 2013 [1]. The consequences of such a large uninsured population are great and affect those individuals who do not purchase health insurance, as well as the rest of society, through cost shifting, increased insurance premiums, and higher taxes. This is the major reason for the Obama administration’s urgency in passing health insurance reform in 2010.

The existing literature clearly reveals that the uninsured rate varies by age. Young adults represent the largest percentage of Americans who are not covered by a health insurance plan [2]. Young adults who are between the ages of 18 and 24 are about 30% less likely to be insured than the rest of the population [3]. The chances of being insured increase as a person grows older. About 25% of people between the ages of 25 and 34 are uninsured compared to 18% between the ages of 35 and 44 and 13% between the ages of 45 and 65.

The major aim of the present study is to determine why so many young adults lack private health insurance coverage by closely examining the demographic and perceptual variables that affect a young adult’s opportunity to become insured. Perceived health status, perceived need, perceived value, socioeconomic status, and other demographics have been identified in the existing literature as determinants of health insurance status for both young adults and the American population in general. Structural equation modeling is used to examine the impact of these variables on health insurance coverage.

The landscape of the health insurance industry was changed by the passage of the Affordable Care Act (ACA) in March 2010, and the Supreme Court’s June 2012 decision to uphold the individual mandate for health insurance coverage associated with it. The ACA now enables young students to stay on their parents’ insurance plans until they are 26 years old, which will allow more young adults access to necessary health care services. For a variety of reasons, many young adults currently choose not to purchase health insurance when they turn 18 or when they graduate from college. Furthermore, in 2014 the ACA allowed for an expansion in Medicaid. This allowed adults below 133% of the federal poverty level to be insured by Medicaid, covering approximately 7.1 million more adults than were previously covered by health insurance [4].

Premium subsidies will conceivably cover another 6 million young adults [4]. Young adults above 133% of the poverty level will be eligible for subsidized health insurance coverage. The passage of the ACA creates penalties for individuals who do not purchase health insurance if they qualify to do so. Penalties for opting out will vary year to year but will reach a maximum of $695.00 or 2.5% of an individual’s income, whichever is less, by the year 2016 [5]. Young adults who do not have the luxury of being covered by their parents’ plans or who will not qualify for subsidized plans will still be required to purchase health insurance or risk a penalty. Young adults will be forced to pay this penalty if they refuse coverage and are qualified for a health insurance plan that offers coverage premiums for less than 8% of their income.

In Massachusetts, where health insurance has already been mandated with tax penalties for those who do not purchase it, the majority of young adults have purchased a plan. Despite this, young adults are still the number one demographic group least likely to be insured in the state [5]. The mandate for health insurance in Massachusetts has shown that including more people in the risk pool, while in turn lowering health insurance premiums, increases the likelihood that a young adult will purchase health insurance.

An analysis of why young adults do not purchase health insurance may provide insight into whether young adults will choose to purchase insurance or pay the penalty when the ACA is fully implemented. Will young individuals across the country follow this trend of not purchasing health insurance? It is assumed that various subsidies will lower the cost of insurance for many youth, thus removing one impediment to being insured. It is also assumed that the subsidies will significantly increase the proportion of insured young adults; perhaps national trends will follow the trends in Massachusetts. Further research is needed to predict how young adults will act across the country.

The present study examines determinants of health insurance coverage for young adults by measuring respondents’ perceptions of their health status, need for healthcare services, and value of such services. We examine the impact of traditional demographic factors typically studied in previous research. Structural equation modeling, a powerful empirical technique, is used to determine the relationships between variables. Structural equation modeling is appropriate for situations in which one desires to measure the influence of several different factors on one dependent variable, such as the different determinants of health insurance coverage for population subgroups. One advantage of using structural equation modeling in empirical studies is that it allows for the inclusion of latent variables. The inclusion of both latent variables and directly measured variables sets this study apart from other health insurance studies.

### Conceptual framework

Kahneman and Tversky’s prospect theory postulates that the immediate financial investment associated with the purchase of a product (health insurance in this case) is a major factor when deciding whether to purchase it or not [6]. Other decision theories take into account the probable financial loss that may come with a serious illness or injury down the road. Prospect theory, however, allows one to consider that the certain loss associated with paying a monthly premium and upfront costs, rather than the possibility of a major medical bill in the future, is the major deciding factor when it comes to the purchase of health insurance by young adults. An important study outlines the main argument in prospect theory: when it comes to deciding among potential gains, people avoid risk, but when it comes to potential losses, people are risk seeking [6,7]. As the amount of loss associated with a decision increases, it is less likely that an individual will choose to accept that loss.

While prospect theory is useful in understanding how young adults make decisions concerning health insurance, we argue that this conceptual framework should not be applied in isolation. The social ecological model developed by Stokols proposes that “behaviors are influenced by intrapersonal, socio-cultural, policy, and physical environmental factors. These variables are likely to interact, and multiple levels of environmental variables are described that are relevant for understanding and changing health behaviors” [8]. In the context of this situation, the lack of certain environmental resources (e.g., money, education) may prevent individuals from seeking necessary health care services or the insurance to pay for them. In the same context, other environmental factors may prevent insurance adoption.

There are four levels of determinants of health behavior in the social ecological framework: individual, organization, community, and population. At the individual level, a person’s behavior is influenced by their knowledge of risks associated with not having health insurance and their individual income (socioeconomic status and demographics). Socioeconomic status also comes into play at the organizational level. Whether or not one is employed theoretically plays a role in one’s decision-making process. Further, whether or not part-time employment opportunities allow for insurance adoption may be a factor. At the community level, social norms and beliefs influence behavior (perceived need). At the population level, perceived value is one variable that dictates who chooses to be covered by health insurance and who does not.

Perceived health status, perceived need for health insurance, perceived value of health insurance, socioeconomic status, and other demographics all have a theoretical effect on individual health insurance coverage according to the structural equation model for the study. The main variables of interest in this study (perceived need, perceived value, and socioeconomic status) will be examined through the lens of prospect theory. The model used in this study is consistent with prospect theory in that it measures whether socioeconomic status and the price of insurance have more influence on the decision to purchase health insurance than perceived health status, perceived need, and perceived value.

The social ecological model explains that personal behaviors are influenced by a number of different factors that interact with each other. This provides a framework that illustrates that the different variables included in this study are likely to interact with each other on different levels [8]. The final model used in this study was based on a combination of published literature and the two major theories discussed in this section. These two theories fit well together and, when combined, form a theoretical framework that helped guide our study. Prospect theory outlines why individuals make certain choices, and the social ecological model explains how different societal variables interact with each other and affect the individual [8].

### Literature review

#### Actual and perceived health status

There is a popular belief that young adults do not purchase health insurance because they generally experience very good health. Although many young adults are indeed healthy, those with disabilities and chronic illnesses need significant medical care. If these individuals do not have health insurance, the consequences can be deadly. Young Americans with disabilities and long-term health problems usually have private or public health insurance coverage through their parents [9].

Once young adults turn nineteen, they are faced with enormous challenges when it comes to purchasing health insurance unless they are students and still covered by their parents’ insurance plans [9]. Many think that Medicaid acts as a safety net for all people with disabilities; however, this is not always the case. There is a growing number of young adults with long term health problems who cannot obtain private health insurance and cannot be covered by Medicaid because they are not categorized as being functionally disabled [9]. This means that there are sick individuals in our country (young and old) who cannot work and are left with limited or no options for purchasing health insurance.

One study conducted in 2000 did not find a strong association between health status and health insurance coverage [10]. However, another study in 2003, comparing two timely national surveys, found that the chance of purchasing private health insurance coverage is approximately 50% *lower* for individuals who are in fair or poor health compared to individuals who consider themselves to be in excellent health [11]. The researchers also found that premiums are actually 13 to 16% higher for individuals who experience moderate health difficulties and 43 to 50% higher for individuals that experience major health difficulties compared to individuals who are in excellent health.

Americans with private health insurance are very healthy because those in good health are generally able to afford coverage [12]. This conclusion was reached after examining data collected from the Medical Expenditure Panel Survey, which showed that being in good health is associated with a greater chance a person will purchase health insurance. The U.S. Census Bureau found, using 2001 data from the Survey of Income and Program Participation, that those in excellent health had health insurance at higher rates than those in poorer health.

#### Perceived need

One reason that some younger adults do not purchase health insurance may be that they feel immortal or invincible to serious illnesses or injuries, especially young adult males [13]. It is certainly not difficult to understand that this feeling of invincibility may be a contributing factor to a lack of health insurance coverage among young adults. Many young adults simply do not seek out regular care, do not fully appreciate health insurance, and decide to spend their income on rent, food, and transportation [13].

Generally, young adults are healthier and in better shape than older adults, and they heal more quickly from injuries than older adults. However, this does not necessarily mean that they place no value on health insurance.

Some young adults may not recognize the importance of being insured or not see it as a benefit [14]. Many young adults have not yet had a serious medical problem and do not see the possibility of a serious injury in their near futures. While some young adults may realize that there are risks involved with being uninsured, they are typically willing to accept the risks.

Contrary to popular belief, however, many young adults *do* consider health insurance to be important [15]. As the author of the Biennial Health Insurance Survey explains, when questioning young workers about their desire for health insurance, seven out of ten of those between 19 and 29 years of age said that health insurance was very important to them in deciding whether to take a job, a rate similar to that for older workers (p. 5). The survey found that 71% of young adults with jobs actually accept health insurance. Furthermore, it is reported that 70% of young adults believe that the existence of a company health insurance plan is an important factor when deciding whether to take a job [16]. However, only 42% of young adults that are employed are covered by a health plan sponsored by their employer, compared to 62% of working older adults. One possible explanation for this low rate of insurance uptake among young adults is that they are more likely to be employed on a part-time or temporary basis by an employer that simply does offer health benefits [16]. These studies raise questions about the conventional wisdom that young adults feel that they do not need or want health insurance.

#### Perceived value

Previous literature indicates that people choose to invest in health insurance if the perceived benefits exceed out-of-pocket costs. In many instances, a perceived value is assigned to having health insurance. The lack of affordability is a significant reason why people do not purchase health insurance, and it is argued by Monheit (2008) that some people prefer a steady income with no health insurance coverage over an unquantifiable value associated with having health insurance (i.e. the perceived value does not outweigh the cost of coverage) [17].

The rising cost of health insurance is a major reason why so many young adults do not purchase health insurance. Approximately 50% of respondents to a Humana health insurance survey indicated that their primary reason for not buying health insurance is that they cannot afford it [13]. The price of health insurance is number one on the list of explanations for why young adults choose to be uninsured [14]. A study by Markowitz et al indicated that 40% of the uninsured population between the ages of 18 and 24 stated that expense was the primary reason for not being covered by health insurance [18]. This reason ranked as the highest percentage among socioeconomic status, demographics, and health status categories. Their study’s variables closely resemble those examined in the present study and illustrate the importance of including a variable related to perceived value in any health insurance study.

The Henry J. Kaiser Family Foundation also reports that the number one reason people of any age are uninsured is the high cost of health insurance in the United States [19]. The Wisconsin Department of Health and Family Services found that the high cost of health insurance prevents young adults in Wisconsin from purchasing health insurance as well [20]. Specifically, 67% of the young adults who participated in the Wisconsin study said they could not purchase health insurance because they were simply unable to afford it.

#### Socioeconomic status

While many different issues contribute to a person’s lack of health insurance coverage, it is clear from the literature that having a low income is one reason why individuals are not willing to purchase health insurance plans [21]. Socioeconomic status can be measured by multiple variables including educational attainment. The literature shows a positive relationship exists between years of education and socioeconomic status with health insurance coverage. Those that have the least education are nearly five times less likely to have health insurance than more highly educated peers [22].

Individuals with incomes below the poverty level are two times less likely to be insured [23]. The Kaiser Commission on Medicaid and the Uninsured reports that low-income adults are at a substantial risk of not having health insurance and make up about 50% of the uninsured population [24]. In other empirical research, a positive relationship was found between age, race (Caucasian), income and employment with insured status [25].

Research in 2003 analyzed the 1997 and 1999 National Survey of America's Families (NSAF) to determine what financial difficulties adults without health insurance face [21]. The study reported that “it was found that over 40% of all adults in the sample reported food, housing, or health care hardship over the past year” [21] and that “overall, 38% of moderate and higher-income uninsured adults and 70% of low-income uninsured adults were not able to afford health insurance because they struggle with paying for food and housing” [21]. Socioeconomic status appears to play an integral role in whether or not a person of any age purchases health insurance.

#### Demographics

Any discussion of demographics and health insurance status should include an examination of minorities. The Kaiser Commission on Medicaid and the Uninsured Report explained that minorities make up approximately 34% of the non-elderly population but 52% of the uninsured population [24]. Other studies also indicate that minority status has a negative relationship with health insurance coverage.

Members of minority groups have a higher likelihood of being uninsured, and this is particularly true if they have incomes that are at or below the federal poverty level [26]. One researcher stated that “young African American men are the least likely to have health insurance” [14] (p.5). In addition, Hispanics are more likely to be uninsured than other racial and ethnic groups [27]. Even though a high number of white young adults (31%) are uninsured, they are still more likely to be covered by some sort of health insurance plan than African Americans or Hispanics.

Another researcher stated that young men have a greater likelihood of not having health insurance than young women [14]. Overall, young men have the lowest rate of health insurance coverage. Historically, rates of health insurance coverage for young men have been lower than the corresponding rates among older men, but the gaps in coverage have grown wider in recent years [28].

Researchers have discovered that Caucasian individuals and women in general have health insurance at rates higher than others [29]. The National Center for Health Statistics (2003) supported this finding and reported that women are more likely to have health insurance.

In summary, previous research indicates that actual and perceived health status, perceived need, perceived value, socioeconomic status, and demographic factors are all related to an individual’s health insurance status. The present study will more specifically aim to determine the degree to which they are predictors of the health insurance status of young adults.

#### Hypotheses

The following hypotheses are based on the established conceptual framework and the results of the previously discussed literature. These hypotheses guided our study of the health insurance status of American youth:

**H1**: An individual’s perceived health status is negatively related to the likelihood of being insured.

**H2:** An individual’s perception of health insurance as valuable (worth the cost) is positively related to the likelihood of being insured.

**H3:** An individual’s perception of a need for health insurance is positively related to one’s likelihood of being insured.

**H4:** An individual’s socioeconomic status is positively related to the likelihood of being insured.

## Methods

### Data source

The public data used in this study comes from the 2005 and 2008 Medical Expenditure Panel Survey (MEPS). The MEPS collects information on the health services that people use, how often they use those services, how much those services cost, and how those services are paid for. Important to this study, the survey also collects data on the “the cost, scope, and breadth of health insurance held by and available to U.S. workers” [30]. This public data was accessed through the MEPS Data section of the Agency for Healthcare Quality and Research website. Archival data was retrieved by downloading the entire dataset for the 2005 and 2008 household component in the form of an SPSS software file, allowing for data analysis, manipulation, and imputation with statistical software.

### Sampling

For this study, publicly available data from the household component of the MEPS was used. This section of the survey is used to gather data from a sample of families and individuals in selected communities across the United States. The sample consists of a subsample of households that answered questions on the National Health Interview Survey. The sample comes from the civilian non-institutionalized population of the United States provided by the National Health Interview Survey [30]. The four census geographical regions that this sample comes from include the Northeast, South, West, and Midwest. The data obtained from each region is representative of that region. Data was collected using a computerized assisted personal interview (CAPI) method over household telephones.

Ultimately, unweighted data was used in this study, and the study is not a nationally representative study. However, the strength of this dataset is the large sample size and breadth of questions asked in the questionnaire. Overall, the number of individuals surveyed in 2005 was 32,320. For the purposes of this study, the sample size was reduced to 3,326 by excluding participants who were not between the ages of 18 and 24. The sample size was further reduced to 1,340 when listwise deletion was performed; all subjects containing missing values were removed from the statistical analysis. In 2008, 31,262 individuals were surveyed. This sample size was reduced to 3,073 when the sample was narrowed down to young adults between the ages of 18 and 24. Subjects with missing data were removed from the sample, further reducing the sample size to 1,463 subjects. We acknowledge that this age subgroup is not completely homogenous and that many 18 year olds were eligible for health insurance coverage under their parents’ plans at the time of data collection. While there certainly was a difference in insurance rates between 18 years olds and the rest of the subgroup, we did not find that the difference was large enough to exclude individuals that were 18 years old from the study.

Comparisons were made between the subjects included in the study and the subjects not included in the study because of missing data. A statistical t-test and chi-square tests were used to determine the difference between data that was used in this study and the sample obtained from the MEPS Data. It was determined that subjects with some missing data were less educated, less likely to be covered by private insurance, and more likely to be members of minority ethnic groups. Since there were still large numbers of participants in all subgroups, relationships between variables can still be studied using structural equation modeling and those relationships will still have some validity [31].

### Variables

**Figure 1:**
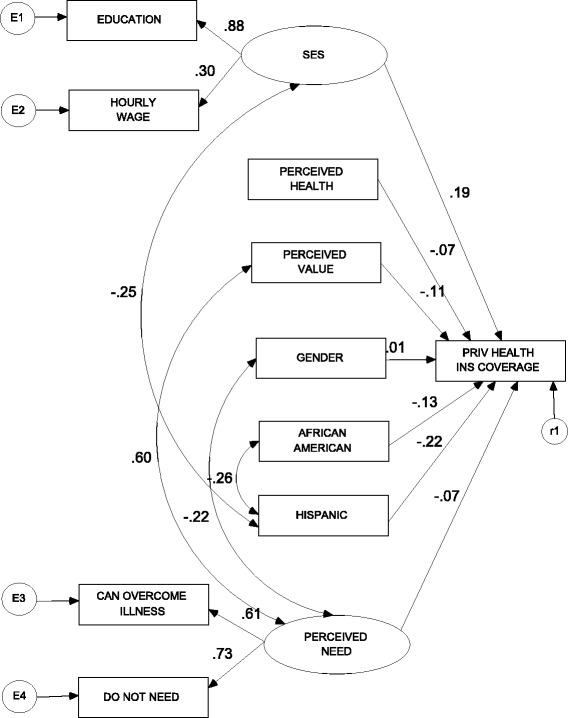
Regression Coefficients for Revised Model of Demographic and Perceptual Determinants of Private Health Insurance Status among Young Adults (2005).

**Figure 2:**
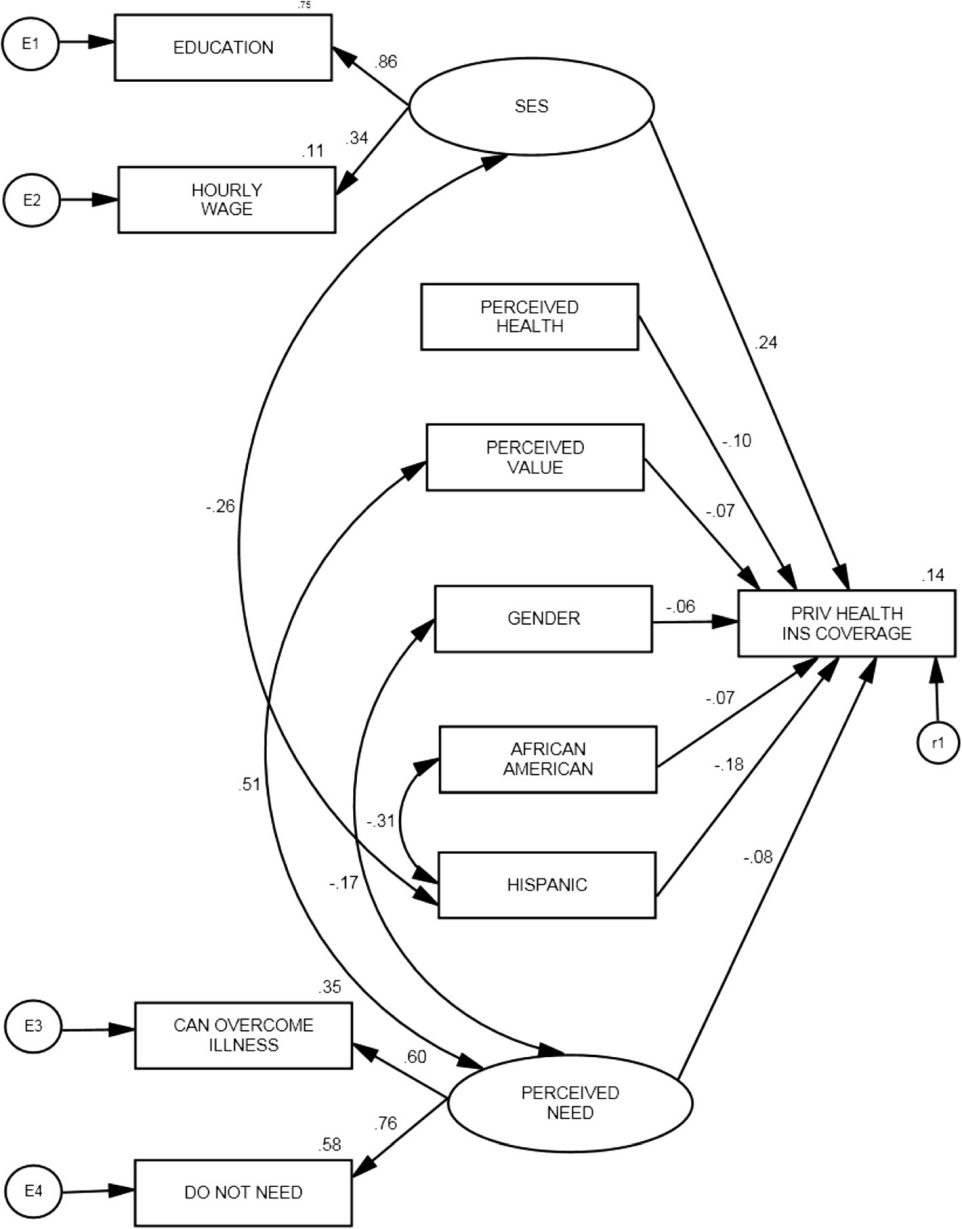
Regression Coefficients for Revised Model of Demographic and Perceptual Determinants of Private Health Insurance Status among Young Adults (2008).

The Table of Operational Definitions and Means (Table 1) correlates with the independent and dependent variables of study in Figures 1 and 2. All variables can be found in archival data collected using the MEPS. Private Health Insurance Coverage was the only dependent variable in this study. This variable measured whether or not an individual was covered by a private health insurance plan at the time of the survey. The independent variables included Perceived Health Status, Perceived Need, Perceived Value, Socioeconomic Status, and Demographics (Gender, African American, Hispanic). Because of the extensive literature pertaining to demographics and health insurance coverage, the demographic variables were used as control variables. While variables that might measure perceived need exist in the MEPS data, the authors strongly felt that ‘can overcome illness’ and ‘do not need health insurance’ most effectively captured the perceived need construct.

**Table 1:** **Operational Definitions of Variables and Means/Percentages for All Variables**

**Variable**	**Description**	**Type**	**Codes**	**Mean or Percent 2005 | 2008**
Private health insurance coverage	Whether a person is covered by a private health insurance plan	Dependent	1 = Yes	52.1 | 49.2
			0 = No	
Perceived health status	Health status according to individual	Independent	1 = Excellent	36.5 | 38.6
			2 = Very Good	32.9 | 32.9
			3 = Good	24.6 | 22.1
			4 = Fair	5.4 | 5.7
			5 = Poor	0.5 | 0 .5
Can overcome illness	Can overcome illness without medical help (Measures Perceived Need)	Independent	1 = Disagree Strongly	28.6 | 28.5
			2 = Disagree Somewhat	23.3 | 24.8
			3 = Uncertain	17.3 | 15.7
			4 = Agree Somewhat	24.3 | 25.1
			5 = Agree Strongly	6.5 | 5.9
Do not need	Do not need health insurance (Measures Perceived need)	Independent	1 = Disagree Strongly	44.9 | 45.3
			2 = Disagree Somewhat	21.2 | 21.5
			3 = Uncertain	13.8 | 12.6
			4 = Agree Somewhat	15.4 | 16.3
			5 = Agree Strongly	4.8 | 4.3
Perceived value	Health insurance not worth cost	Independent	1 = Disagree Strongly	36.3 | 33.4
			2 = Disagree Somewhat	20.6 | 21.6
			3 = Uncertain	19.6 | 17.8
			4 = Agree Somewhat	15.1 | 16.8
			5 = Agree Strongly	8.4 | 10.4
Education	Individual education level (Measures SES)	Independent	1-8 = Grades1-8	2.9 | 2.9
			9-11 = Grades 9-11	24.8 | 18.2
			12 = Grade 12	36.1 | 38.6
			13 = 1 Year College	13.1 | 12.3
			14 = 2 Years College	10.4 | 13.1
			15 = 3 Years College	5.4 | 7.0
			16 = 4 Years College	4.9 | 6.4
			17 = 5+ Years College	1.3 | 1.4
Hourly wage	Hourly wage (Measures SES)	Independent	Indicated by continuous dollar values	9.06 | 10.67
Gender	Gender of individual	Control	0 = Male, 1 = Female	50.00 | 50.5
African American	African American	Control	0 = White, 1 = African American	15.1 | 19.5
Hispanic	Hispanic	Control	0 = White, 1 = Hispanic	27.3 | 32.3

It is important to note that variables exist within the 2005 and 2008 MEPS data related to student status, whether individuals were living in a household with their parents or not, and whether an individual’s employer offered health insurance. Structural equation models including these variables were initially created, but these variables were not found to be significant in 2005 or 2008 and therefore not presented in the present study.

### Statistical methods – structural equation modeling

Structural equation modeling (SEM) was used in order to determine the relationship between the dependent variable in the study (Private Health Insurance Coverage) and the independent variables in the study. SEM is a regression analysis technique that helps to identify relationships in the structural equation models between the dependent and independent variables. A model was constructed and revised based on model fit results using the AMOS 7.0 software package for SEM. The final study model created after revisions indicates the strength of these statistical relationships (Figure 1).

The decision to use SEM over standard logistic regression is based on a number of reasons. Firstly, when the phenomena underlying research questions and hypotheses are complex and multidimensional, SEM is an analysis tool that allows complete and simultaneous tests of all hypothesized relationships [32]. Secondly, a single structural equation model can combine the strengths of both multiple regression and confirmatory factor analysis [33]. Thirdly, SEM is primarily a confirmatory analysis as it allows variables to be initially grouped into theoretically supported constructs. As a result, it is possible to conduct theory-based hypothesis testing. Unlike most other multivariate procedures, SEM provides precise estimates for measurement errors [34]. This is critical in avoiding inaccuracies, particularly when the errors are significant.

Unlike most other multivariate analyses that only focus on observed variables, SEM has the capacity to simultaneously analyze both observed and unobserved (latent) variables [35]. Hypothesis testing for fit, as mediation, is best conducted through path analysis [36]. SEM incorporates path analysis through its structural model.

SEM also allows the researcher to ask “new questions that could not have been addressed without the technology and thinking that underlie SEM” [37]. The use of SEM allowed for the determination of variables that most affect a young adult’s decision to purchase health insurance. Moreover, this method allowed us to determine how different variables interacted with each other.

With regression analysis incorporated, SEM goes a step beyond normal regression. SEM has the ability to specify latent variable models that provide separate estimates of relationships between latent constructs and their indicators, and between constructs [31]. Standard regression does not allow for the analysis of latent variables. The value-added component of using latent variables is that it allows for underlying issues to be explored, while variables that are related to each other can be grouped together. Using latent variables allows researchers to assess psychometric properties in order to interpret such variables. It also allows the researcher to hypothesize and test causal relationships that potentially exist in the real world and loosens the data constraints that multiple regression imposes. Due to the aforementioned advantages and focus on perceptual variables in this study, SEM was the preferred methodology for the present study.

### Assumptions Required When Using Structural Equation Modeling

A large part of the SEM technique is the fit of the data within the model. If the sample data does not fit the model, then the model results are not reliable. When the data fits the model, multivariate normality is assumed [31]. Reaching the point where the data fits the model is an iterative process that may require dropping cases and correlating variables. SEM can provide measures of global fit that can “provide a summary evaluation of even complex models that involve a large number of linear equations” [31]. If the data fits the model, SEM allows for research to be replicable. SEM is one of the most “broadly applicable statistical procedures currently available” [31].

A weakness of SEM is that it is often necessary to systematically reduce the sample size or omit variables to get the data to fit the model [31]. Many researchers claim that this weakness is overcome by including residual terms that show the composite effects of the unmeasured influences on variables. Our model includes residual terms for each variable included, in order to mitigate this weakness.

Standardized regression coefficients and outputs obtained after running the data through the model provided us with the necessary information needed to revise and later accept our model. A close examination of indicator statistics and modification indices provided by the AMOS 7.0 software package allowed us to make necessary changes to the model in order for it to fit the data. After a final analysis, output obtained from the AMOS 7.0 software program was analyzed to determine how well the indicators included in the data explained health insurance coverage among young adults.

## Results

The values for each variable studied in 2005 and 2008 are provided in Table 1. The Perceived Health Status variable is an observed construct. The values for the variable Private Health Insurance indicate whether the person responding to the MEPS was covered by private health insurance or not. The Perceived Need variable is a latent construct and was measured by the following two variables: 1) Can Overcome Illness and 2) Do Not Need Health Insurance. The Perceived Value variable is an observed construct. This variable was measured by determining to what degree the respondent agreed or disagreed that health insurance was not worth the cost.

Socioeconomic Status is a theoretical construct that was measured by: 1) Hourly Wage and 2) Education Level. The next variables are related to gender and ethnicity. A review of other studies, using structural equation modeling as a method of statistical analysis, revealed that using one race as a reference category and using another race as a dummy variable was well suited to running a structural equation model. Consequently, dummy code variables are used for examining the different demographic variables in this study. The dummy code for being African American showed the unique effect of being African American versus being White; the dummy code for the Hispanic variable showed the unique effect of being Hispanic versus being White.

Figures 1 and 2 show the final structural equation models for study years 2005 and 2008, respectively. Correlations between variables are noted on each arrow using the same dependent and independent variables that were introduced in the Table 1. As mentioned earlier, the nature of SEM requires that the data fit the model before continuing on with the analysis. An initial run of the data and analysis of the “goodness of fit” statistics showed that a better fit could be achieved by making model adjustments. A close examination of indicator statistics and modification indices provided by the AMOS 7.0 software package allowed us to make necessary changes to the model required by the “goodness of fit” statistics. These changes consisted of correlating variables with very large modification indexes.

Table 2 shows the factor loadings for the year 2005. This table was used to draw conclusions regarding the variables of the study. As mentioned previously, SEM employs the use of regression. Regression of the variables in the final model (Figure 1) with the Private Health Insurance Coverage variable yielded the following factor loadings associated with having coverage: (PERCEIVED HEALTH = -.072), (PERCEIVED NEED = -.066), (PERCEIVED VALUE = -.110), (SES = .185), (GENDER = -.006), (HISPANIC = -.226), (AFRICAN AMERICAN = -.127). The variables SES (Socioeconomic status), HISPANIC, and AFRICAN AMERICAN were significantly related at the .001 level to the variable PRIV HEALTH INS COVERAGE. The other relationships in the model were not significant.

**Table 2:** **Revised SEM Results for the Effects of Independent Variables on Private Health Insurance Coverage for the Year 2005**

			**Unstandardized estimates**	**S.E.**	**C.R.**	**P**	**Standardized estimates**
PRIVATE HEALTH INSURANCE COVERAGE	<---	PERCEIVED HEALTH	-.038	.014	2.825	.005	-.010
PRIVATE HEALTH INSURANCE COVERAGE	<---	PERCEIVED NEED	-.041	.029	-1.403	.161	-.08
PRIVATE HEALTH INSURANCE COVERAGE	<---	PERCEIVED VALUE	-.041	.014	-2.875	.004	-.07
PRIVATE HEALTH INSURANCE COVERAGE	<---	SES	.071	.014	5.230	***	.24
PRIVATE HEALTH INSURANCE COVERAGE	<---	GENDER	.006	.027	.225	.822	-.06
PRIVATE HEALTH INSURANCE COVERAGE	<---	AFRICAN AMERICAN	-.175	.037	-4.742	***	-.226
PRIVATE HEALTH INSURANCE COVERAGE	<---	HISPANIC	-.252	.034	-7.332	***	-.18

***indicates statistical significance at p < .001 level.

Table 3 shows the factor loadings for the year 2008. Regression of the variables in the final SEM model (Figure 2) with the Private Health Insurance Coverage variable yielded the following factor loadings: (PERCEIVED HEALTH = -.010), (PERCEIVED NEED = -.08), (PERCEIVED VALUE = -.07), (SES = .24), (GENDER = -.06), (HISPANIC = -.226), (AFRICAN AMERICAN = -.18). The variables SES, HISPANIC, AFRICAN AMERICAN, PERCEIVED NEED, PERCEIVED HEALTH, and PERCEIVED VALUE were significantly related at the .001 level to the variable PRIV HEALTH INS COVERAGE.

**Table 3:** **Revised SEM Results for the Effects of Independent Variables on Private Health Insurance Coverage for the Year 2008**

			**Unstandardized estimates**	**S.E.**	**C.R.**	**P**	**Standardized estimates**
PRIVATE HEALTH INSURANCE COVERAGE	<---	PERCEIVED HEALTH	-.054	.013	-4.166	***	-.102
PRIVATE HEALTH INSURANCE COVERAGE	<---	PERCEIVED NEED	-.049	.025	-1.962	***	-.075
PRIVATE HEALTH INSURANCE COVERAGE	<---	PERCEIVED VALUE	-.026	.011	-2.259	***	-.071
PRIVATE HEALTH INSURANCE COVERAGE	<---	SES	.065	.010	6.763	***	.243
PRIVATE HEALTH INSURANCE COVERAGE	<---	GENDER	-.061	.025	-2.398	.822	-.061
PRIVATE HEALTH INSURANCE COVERAGE	<---	AFRICAN AMERICAN	-.084	.033	-2.585	***	-.067
PRIVATE HEALTH INSURANCE COVERAGE	<---	HISPANIC	-.195	.029	-6.721	***	-.183

***indicates statistical significance at p < .001 level.

## Discussion and conclusions

### Perceived and actual health status

Perceived health status was not found to have a statistically significant relationship with private health insurance coverage in 2005. However, in 2008, perceived health status was found to have a statistically significant relationship with health insurance coverage. These findings, associated with the first hypothesis, replicated the variation that exists in the literature regarding the perceived health status’s impact on health insurance coverage. Some of the previous research indicates that health status plays a role in a person’s decision to purchase health insurance while other studies do not. While there are multiple studies focusing on the relationship between health status and health insurance coverage, there is a lack of literature examining the relationship between perceived health status and health insurance coverage.

Further study is needed to determine the precise relationship between perceived health status and health insurance coverage.

### Perceived need

Interestingly, the present study of 2005 data indicated that perceived need (Hypothesis #2) did not significantly impact health insurance coverage among young adults in the sample. Conversely, the 2008 findings illustrated that perceived need did significantly impact young adult health insurance coverage among those surveyed. As presented in the literature, some studies show that young adults do not purchase health insurance because they do not believe they need it. Other studies claim that young adults do see the value in health insurance but simply cannot afford it [15,16].

Researchers explain how health insurance companies often believe that young adults do not have a need for routine health care, and do not see the value in or appreciate health insurance [13]. Conversely, some young adults simply do not understand the value of health insurance because they have not yet had a need for it [14]. The Joint Economic Committee reports that most young adults are usually healthy and believe that the cost of health insurance is greater than their expected risk [3]. Conversely, a 2006 study contends that young adults do not easily dismiss the risks of not having health insurance, and found that 70 percent of young adults do indeed regard health insurance as an important factor when choosing employment [15].

The results of our study suggest that socioeconomic status may have overwhelmed the effect of perceived need in decision making in the 2005 sample. Perhaps, even though the young adults in the sample believed that they could overcome illness or injuries without medical help and that they did not believe in health insurance, these were not the deciding factors in whether to purchase health insurance.

### Perceived value

In reference to the third hypothesis, our results showed that within the samples, individuals’ perception of health insurance’s value (worth or not worth the cost) was not significantly correlated with the likelihood of having health insurance in 2005, but perceived value was a statistically significant variable in 2008. The literature and conventional wisdom dictates that if a person cannot afford health insurance, they cannot purchase it. This is consistent with the study findings for 2008 regarding the Perceived Value variable.

Other researchers claim that the expense of health insurance is the primary reason for young adults not being covered by health insurance [14,18]. The results of the present study were consistent but not confirmatory of these two studies. Similarly, the Agency for Healthcare Research and Quality (AHRQ) and the Henry J. Kaiser Family Foundation explained that the main reason people do not purchase health insurance is cost [19,30].

### Socioeconomic status

The results of our structural equation analysis showed that socioeconomic status did indeed influence whether a young adult was covered by private health insurance in both the 2005 and 2008 samples. This confirms the fourth hypothesis. Within the samples, as socioeconomic status increases, the likelihood of purchasing private health insurance increases. While not unexpected, this is an important finding of the study and supports the results of previous research that socioeconomic status predicts health insurance coverage. This study supports the results of the Indiana Family and Social Services Administration (2002) study, which found the lowest rates of insurance among those with lower incomes and among African Americans.

The U.S. Census Bureau found that the probability of being insured did increase with a higher income and more education in 2002 [1]. Similarly, a 2004 study claimed that income and education have a positive effect on a person having health insurance [38]. The Congressional Budget Office reported that people with the least education are five times more likely to be uninsured [22]. The Kaiser Commission on Medicaid and the Uninsured reported that low-income adults are at a higher risk of being uninsured than the rest of the population [24].

The author of a 2003 study argued that socioeconomic status appears to play a role in whether or not a person purchases health insurance [21]. In 2004, it was also found that the strongest relationship between health insurance coverage determinants and health insurance coverage existed between income and having health insurance [25]. The findings of the present study of young adults are consistent with the results of the above studies of the general population that socioeconomic status predicts health insurance coverage.

### Demographics

The examinations of being African American versus being White and being Hispanic versus being White revealed a statistically significant relationship in both the 2005 and 2008 samples. It was discovered that, when compared to African Americans and Hispanic people in the sample, White people are more likely to have health insurance. While not surprising, it is important to note that these variables, along with the Socioeconomic Status variable, were the only variables found to have a statically significant relationship with the Private Health Insurance variable.

The literature suggests that demographics do have an impact on a person having health insurance. The Kaiser Commission on Medicaid and the Uninsured showed that minorities make up the majority of the uninsured population [24]. Studies report that members of minority groups are more likely to be uninsured than other members of society [26] and that African American men are the least likely to have health insurance [14]. Another study also reported that being African American reduced the probability of being covered by health insurance [39].

The U.S. Department of Labor reported that women are more likely to purchase health insurance than men [40]. Similarly, a study found that females were more likely to have private health insurance [29]. More recent studies also found higher levels of health insurance coverage among females than males [27,39].

While the results of this study showed that, within the sample, being either African American or Hispanic played a significant role in whether or not an individual had health insurance, the results of our study did not show a significant relationship between gender and health insurance coverage in 2005 or 2008. We have speculated that perhaps young adult women differ from the rest of the adult population concerning their decisions to purchase health insurance. Women may become more health conscious as they age.

### Theories examined

Since the Perceived Value construct in the study model did not have a significant effect on a young adult having health insurance in 2008, it cannot be concluded that within our sample prospect theory was a theory that solely drove whether a young adult took up health insurance.

However, socioeconomic status (which is income related) did have a significant relationship with private health insurance coverage in the 2005 and 2008 samples. There is a positive relationship in this instance, meaning that prospect theory may have indeed played a role in young adults’ health insurance uptake within our study sample.

The fact that socioeconomic status, race, and ethnicity all play a strong role in determining who purchased health insurance means that this was affected by several different influences on the individuals in our sample. The results show that there is no one single factor that led to the young adults in our sample purchasing health insurance; it was a combination of several factors. This indicates that in our study sample, Stokol’s socio-ecological model is a theoretical construct that may have played a part in whether or not young took up health insurance.

### Limitations

As in any study, there are limitations to our findings. Since archival data was used, we did not have the luxury of choosing outright which variables to study. Another limitation of this study is that the latent variables, SES and Perceived Need, were measured by only two observed variables. Researchers recommend that at least three indicators be used when measuring a latent variable [41].

The listwise deletion of cases because of missing data is a weakness of this study. A close analysis of both years shows that a large amount of the missing data was due to the fact that some respondents (1) failed to answer the question related to income or (2) failed to answer one or all of the three attitudinal questions (“can overcome illness,” “do not need health insurance,” and “perceived value”). In order to ensure that the study was representative overall, comparisons were made between the subjects that were included in the study and study subjects that were not included because of missing data. These comparisons showed how using complete data affected the composition of the sample. It is important to note that subjects with some missing data were less educated, less likely to have private insurance, and more likely to be members of minority ethnic groups in both 2005 and 2008. More specifically, t-test results showed the equality of means between data that were used in this study and the sample obtained from the MEPS Data. Notably, there was a difference in means between the two groups when education levels were scrutinized in 2005 and 2008. The group with complete data had higher levels of education.

While a t-test is appropriate for comparing the means of two groups of variables that are continuous or categorical, a chi-square test is more appropriate for comparing the means of nominal dichotomous variables, e.g. male/female. A chi-square test comparing the proportion of males and females between the population and the study sample revealed that there was no significant difference between the proportions of males and females. The results of a chi-square test comparing the proportions of African Americans and non-African Americans between the population and the study sample revealed that the study sample with only complete data was less likely to contain African Americans than the population obtained from the MEPS. Similarly, a chi-square test comparing the proportions of Hispanics and non-Hispanics between the population and the study sample revealed that the study sample was less likely to include Hispanics than the population. A chi-square test comparing the proportion of individuals who were covered by private health insurance and individuals who were not covered by private health insurance revealed that there was no difference between the proportion of people who were insured and who were not.

Unweighted data was used in this study, and, while the SEM results are standardized, the study is not a nationally representative study. We wanted the behavior of individual sample members to be observed and therefore designed the present study to include unweighted data. Variables had to be omitted in order for the data to fit the model. This weakness was mitigated by including residual error terms for each latent variable used in the study, indicating that there was a degree of error associated with any given latent variable.

As previously noted, variables related to student status, whether individuals were living in a household with their parents or not, and whether an individual’s employer offers health insurance exist within the 2005 and 2008 Medical Expenditure Panel Survey. Structural equation models including these variables were initially created, and final results mirrored the results presented in this study. These variables were not found be significant in the 2005 or 2008 samples. The inclusion of these variables would have lowered the sample sizes in both years due to missing data and we decided to exclude these variables in order to present models with larger sample sizes. Our model also does not take account of whether it was the young person’s own decision of whether to purchase health insurance, as those covered by their parents’ insurance may have not taken part in the decision-making process.

### Summary

The results of this non-representative study showed that being covered by a private health insurance plan was largely a matter of having a higher socioeconomic status and being a non-minority in the 2005 sample. However, in the 2008 sample, each of the attitudinal variables (perceived health, perceived value, and perceived need) predicted whether subjects carried private insurance. Our findings suggest that among those surveyed, young adults between the ages of 18 and 24 do not represent a unique segment of the population that behaves differently from the rest of the population. When compared to the literature, this study suggests that young adults behave similarly to older adults in terms of health insurance coverage. All population subgroups were affected by their socioeconomic and minority status in the 2005 sample, and all population subgroups were affected by socioeconomic status, minority status, and attitudinal variables in the 2008 sample.

With the 2005 findings related to perceived need in mind, it can be argued that ‘need’ was not a major factor for young adults in this sample when it came to whether they were covered by private health insurance or not. However, ‘need’ was a major factor in the 2008 sample. The 2005 finding related to ‘need’ contradicted many anecdotal arguments and the conventional wisdom that young adults do not purchase health insurance because they feel they do not need it. The 2008 finding related to ‘need’ was consistent with conventional wisdom. More analysis is needed to determine why this difference exists between years.

We speculate that when the economy deteriorates, as it did between 2005 and 2008, the perception of ‘need’ plays a more powerful role in the decision making process. This was a multiyear study, and we chose 2005 because of the availability of the data at the time of the study and the fact that that year represented a point in time before the economic recession. Data from 2008 was chosen because that year represented a point in time when the United States was going through a recession. Both years also predate any discussion or debate pertaining to the Affordable Care Act. A major impetus for this study was the unique opportunity to measure attitudinal variables and make comparisons between these two interesting years while taking advantage of the strength of SEM.

### Implications

The unique contribution of the present study is the use of SEM, which allowed for the examination of latent variables. The relatively recent data also serves to update the literature. These implications should be of interest to anyone concerned with determining why young adults between the ages of 18 and 24 are not covered by health insurance to the same degree as the rest of the population and anyone interested in making predictions about insurance uptake among this age group.

Based on the results of this study and what is known about the factors preventing the general population from enrolling in health insurance, the reasons that young adults do not enroll in health insurance plans may not differ substantially from the reasons the rest of the population chooses to not purchase health insurance. Some may argue that, with the passage of the Affordable Care Act, the problem of the young and uninsured has been adequately addressed. We contend that this new focus on the uninsured may alleviate the insurance problems faced by young adults, but a major research question for the future still exists: *When given the option of paying for health insurance or paying a penalty, will the cost of insurance or perceived need/perceived value of that health insurance dictate a young adult’s choice to purchase health insurance?*

Within our study sample, socioeconomic status and minority status influenced whether or not a person enrolled in a health insurance plan. While direct inferences cannot be made between this study and a study into whether young adults will choose between either a penalty or health insurance, the present non-representative study showed that within the sample a lack of perceived need was not consistently something that prevented young adults from purchasing health insurance. This implies that the young adults we studied did not generally opt out health insurance because they believed they did not need it. This was not consistent with suggestions by other researchers that young adults will automatically choose whichever choice leaves them with the most money in their bank accounts at the end of the month. Further study is needed, and this study does not offer concrete conclusions on who might be likely to pay the penalty after the ACA is implemented.

Our study results suggest that the young adults in our sample might have indeed seen value in health insurance. This view is consistent with previous research [15,16]. The passage of the Affordable Care Act will provide health insurance coverage to the very poor, leaving those in higher socioeconomic subgroups of the population to be studied. A study similar to this, but one that is nationally representative, should be conducted after the Affordable Care Act reforms are fully implemented. In the meantime, the results of the present study may be useful to anyone studying the behavior of young adults when it comes to insurance uptake. It has been argued that healthcare exchanges will require young adults to participate in order to be successful and that not much history or evidence exists to help different stakeholders predict who will enroll in healthcare exchanges [42]. The present study adds evidence in support of the argument that members of this subgroup may see the importance of being covered by health insurance.

## Abbreviations

ACA: The affordable care act
AHRQ: Agency for healthcare research and quality
CAPI: Computerized assisted personal interview
MEPS: Medical expenditure panel survey
NSAF: National survey of American families
SEM: Structural equation modeling
SES: Socioeconomic status
